# Missed opportunities for HIV diagnosis in Mexico City: unveiling the sex gap

**DOI:** 10.3389/frph.2026.1755785

**Published:** 2026-03-03

**Authors:** Nancy Sierra-Barajas, Yanink Caro-Vega, Nancy Ruiz-Dominguez, Ana Fernanda Ramos-Menchelli, Alvaro López-Iniguez, Angelina Silva-Casarrubias, Jessica Mejia-Castrejon, Karen Juarez-Campos, Juan Sierra-Madero, Brenda Crabtree-Ramirez

**Affiliations:** Infectology Department, Instituto Nacional de Ciencias Médicas y Nutrición “Salvador Zubirán”, Mexico City, México

**Keywords:** advanced HIV, AIDS defining events, missed opportunities for HIV diagnosis (MOHD), people living with HIV (PWH), sex disparity

## Abstract

**Background:**

Women in Mexico are not considered a key population for acquiring HIV and are often not perceived at risk by themselves or their physicians. This limited risk perception can delay testing and contribute to advanced HIV disease. We aimed to determine the frequency of missed opportunities for HIV diagnosis (MOHD) in a tertiary center in Mexico City and compare characteristics between men and women.

**Methods:**

We conducted a retrospective observational study using standardized questionnaires administered to adults newly enrolled in care at a tertiary HIV Clinic between 2013 and 2023. MOHD was defined as a healthcare encounter for HIV related symptoms in which diagnosis was not made within 30 days and or the patient attended at least two medical consultations before diagnosis. We described sociodemographic characteristics, the proportion of MOHD, and advanced HIV defined as baseline CD4 < 200 cells or an AIDS defining condition. Comparisons were made by sex, proportions were stratified by age group, and logistic regression identified factors associated with MOHD.

**Results:**

Of 1,332 questionnaires, 619 individuals reported symptoms and sought medical care before diagnosis; 320 (51.6 percent) met MOHD criteria, including 39 women and 281 men. MOHD was more frequent in women than men (67.2 percent vs. 50.1 percent, *p* = 0.03). Advanced HIV was also more frequent among women with MOHD (51.3 percent vs. 33.8 percent, *p* = 0.02). Women had longer symptom duration, more medical consultations, and longer delays from first medical contact to diagnosis. Increasing age (OR 1.01, CI 1.00 to 1.1, *p* = 0.02) and being a woman (OR 2.3, CI 1.21 to 4.52, *p* = 0.01) were independently associated with MOHD.

**Conclusions:**

In this cohort, missed opportunities for HIV diagnosis were common and occurred more frequently among women than men. Women experienced longer delays to diagnosis and higher rates of advanced disease. These findings highlight persistent gaps in timely HIV diagnosis among women.

## Introduction

In 2024, there were 40.8 million people living with HIV (PWH), 53% of whom were young girls and women, and they accounted for 45% of new HIV cases worldwide. Globally, women have twice the risk of acquiring HIV compared to their male partners, and currently, AIDS-related illnesses are the leading cause of death among women aged 15–49 around the world. In Latin America, the AIDS-related deaths have decreased across most affected groups, but increased among women in some countries: Costa Rica, El Salvador, Mexico, Panama, Paraguay, and Peru (1).

Even though one-fifth of PWH cases are women in Latin America, HIV diagnosis and continuum of care in women still face many challenges, particularly in early testing and access to care and treatment (1). Women are not considered a key population (2) in many Latin American countries (3, 4), and as a result, HIV testing and prevention campaigns do not focus on them. Furthermore, several studies in other regions have shown that women are more likely to have a late diagnosis (5), be lost to follow-up (6), and have worse clinical outcomes (7). These findings likely reflect trends seen in Latin America, where limited regional data indicate that women encounter similar obstacles to accessing timely HIV diagnosis and care (2, 4).

According to the Epidemiological Report of the National Center for Prevention and Control of HIV/AIDS (CENSIDA) 2024, in Mexico, approximately 400,000 people live with HIV and 19% of them are women (4). However, pregnancy remains the most consistent context within the national prevention program in which women are systematically included in public HIV screening efforts (4, 8). As a result, women, particularly those beyond reproductive age, have limited opportunities for early HIV diagnosis (4, 8, 9).

In Mexico, up to half of new HIV cases are diagnosed late (10), and evidence shows that inadequate screening strategies contribute to missed opportunities for diagnosis (12, 19). Among women, late presentation is more common in those who are older, unemployed, or living with a stable partner (10). Even when they present with symptoms suggestive of HIV, diagnosis is often delayed (11).

Furthermore, these missed diagnostic opportunities have critical implications, as late diagnosis contributes to worse clinical outcomes such as multimorbidity (13), higher mortality (14), disability (15), intensive care admission (16), risk of new transmissions (17), and increased direct medical costs (18). Building on this evidence, our study incorporates the currently standardized definition of missed opportunities for HIV diagnosis (MOHD) (19) and expands the perspective by comparing men with women. This approach provides a clear understanding of sex-based differences in MOHD, offering insights that can inform more equitable HIV testing and diagnosis strategies.

## Methods

### Study design

We conducted a retrospective observational study using data collected through standardized demographic questionnaires applied to all patients upon enrollment at the HIV Clinic of the Instituto Nacional de Ciencias Médicas y Nutrición Salvador Zubirán (INCMNSZ) in Mexico City. This questionnaire was designed by infectious disease experts in the HIV field.

### Study population and data source

We included newly enrolled patients, with a confirmed HIV diagnosis, who initiated care at the HIV clinic between 2013 and 2023 and completed the demographic questionnaire. Participants were excluded if they did not report symptoms before HIV diagnosis, if they did not report seeking medical attention before enrollment, or if essential information relevant to the study was missing.

All data were derived from the standardized demographic questionnaire administered upon entry to the clinic. This questionnaire has been administered routinely since 2013 and has maintained a consistent structure throughout the study period, from 2013 to 2023. It includes five closed questions to evaluate missed opportunities for HIV diagnosis:

“Did you have symptoms related to HIV that led to HIV serology testing?”
“How long did you experience symptoms before your first positive HIV serology?”
“Did you seek medical attention for these symptoms?”
“What was the date of your first medical consultation for these symptoms?”
“How many medical consultations did you have before receiving your positive HIV serology?”

Although there is heterogeneity in the definition of MOHD, most of the literature defines it using epidemiological criteria (20) which includes individuals at risk of acquiring HIV who present with a medical diagnosis compatible with an HIV clinical indicator and for whom HIV testing, as recommended by local guidelines, was not performed (20, 21). We based our definition on prior studies that use a time-based approach in which a timely suspicion of symptoms and HIV diagnosis should be sought. Most studies define MOHD as occurring when an individual was not screened for HIV in all relevant encounters 30−365 days before HIV diagnosis (19, 22, 23).

From those included in the present study, we divided them into two groups: MOHD and non-MOHD. MOHD was defined as a healthcare encounter due to clinical symptoms related to HIV infection, in which an HIV diagnosis was not made within 30 days of that encounter, and/or the patient had at least two medical consultations before being diagnosed with HIV. Conversely, a non-MOHD was defined as cases where HIV was diagnosed within the first two medical consultations and/or within 30 days of the initial healthcare encounter for HIV-related symptoms (19).

Advanced HIV was defined as having a baseline CD4 count below 200 cells/mm^3^ and/or the presence of an AIDS-defining condition at the time of diagnosis, regardless of the CD4 cell count (20, 24).

### Statistical analysis

Information from all questionnaires completed between 2013 and 2023 was retrieved from the clinic database. We described by sex the sociodemographic characteristics, duration of symptoms before diagnosis (in months), time from first medical contact to HIV diagnosis (categorized in days), the number of medical consultations before diagnosis, and the proportion of individuals with advanced HIV disease. We compared the distribution of these variables within the sex group, using Fisher's exact test and the rank sum test for independent samples.

Additionally, we assessed the proportion of individuals presenting MOHD and those non-MOHD, as well as their sociodemographic characteristics at enrollment by sex. These variables were analyzed to provide a comprehensive overview of the study population. We stratified the proportion of MOHD by age group at diagnosis (<18, 18–29, 30–49, ≥50 years), not age at enrollment, and compared separately for men and women.

We analyzed the factors associated with MOHD using a logistic multivariate regression analysis, fitting by maximum likelihood estimation with manual entry of variables. We calculated the Odds ratio (OR) and 95% confidence intervals (CI) for each factor included in the model. We used SPSS version 27 for the analyses.

## Results

**Figure 1:**
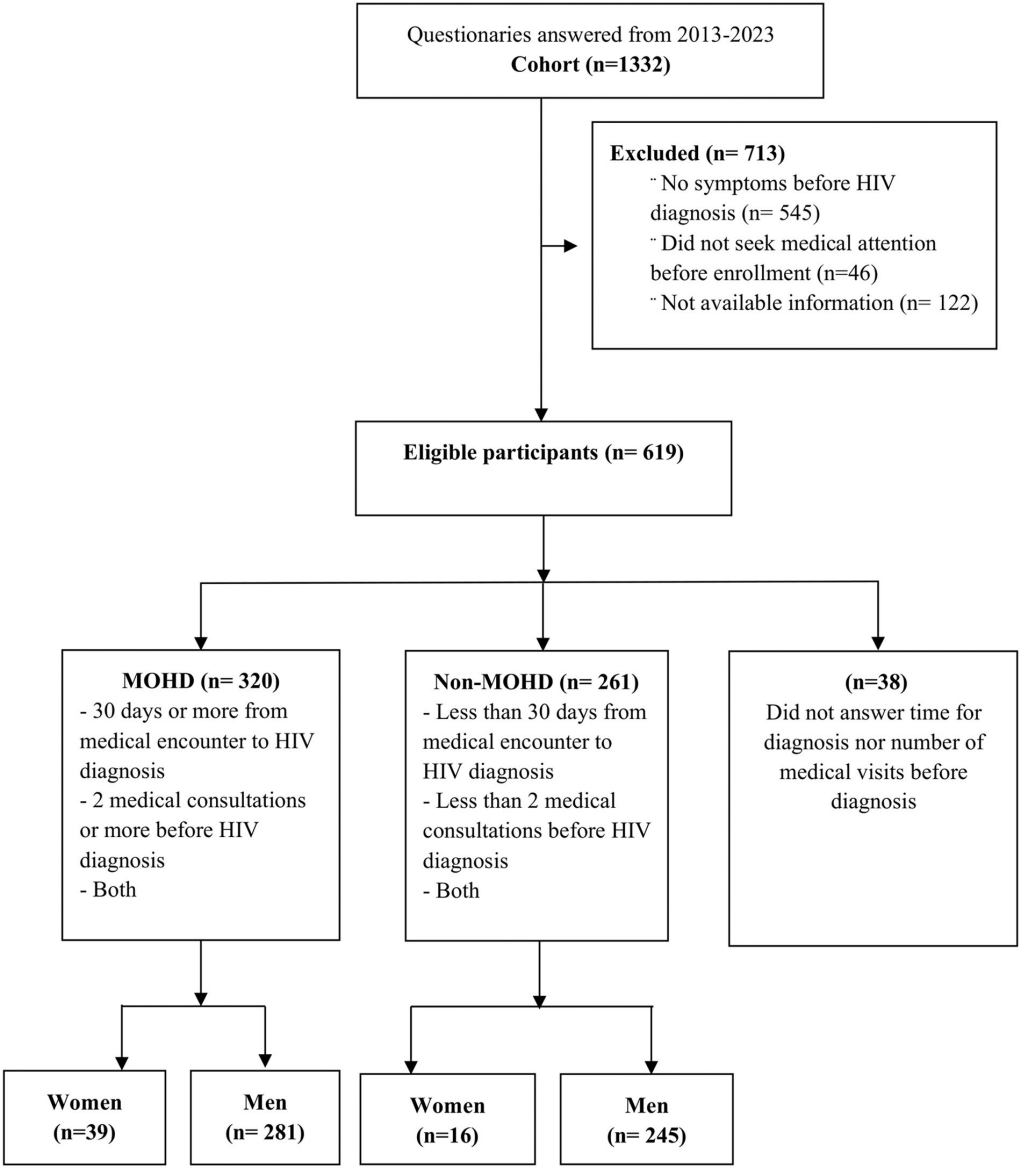
Process for MOHD classification. MOHD, missed opportunities for HIV diagnosis.

Over a ten-year period (2013−2023), 1,332 people responded to the questionnaire; 135 (10.1%) were women and 997 (89.8%) were men. Among these patients, 675 (50.6%) reported having experienced symptoms before their HIV diagnosis but did not seek medical attention, while 619 (46.4%) participants had symptoms and sought medical care before HIV diagnosis. Of them, 320 (51.6%) patients met the MOHD criteria: 39 (12.1%) were women and 281 (87.8%) were men (Figure 1).

Among participants who met the definition of a missed opportunity, 51 (15.9%) individuals experienced a delay of more than 30 days between their initial medical consultation and their HIV diagnosis. Additionally, 130 (40.6%) individuals attended more than two medical consultations for HIV-related symptoms before HIV testing, while 139 individuals met both criteria (43.4%). Among the total of men who sought care for HIV-related symptoms, the proportion of MOHD was 50.1% (281/561); while 67.24% (39/58) of the women with symptoms fulfilled the definition of MOHD.

Baseline characteristics of the included patients are shown in Table 1. Women were significantly older than men [median age: 37 years [IQR 27−48] vs. 32 years [IQR 27−42], *p* = 0.05]. Educational attainment was lower among women, with a median of 12 years of education (IQR 9−14) compared to 14 years (IQR 12−16) in men (*p* = 0.001). Marital status also differed significantly, with 24.6% of women being married compared to 9.7% of men (*p* = 0.001). Employment rates were lower among women, with only 29.3% being employed vs. 50.7% of men (*p* = 0.002) (Table 1).

**Table 1:** Basal characteristics of patients included by sex.

Variable	Men *n* = 561 (90.6%)	Women *n* = 58 (9.4%)	*p* value
Age at enrollment in care, median (IQR)	32 (27–42)	37 (27–48)	0.05
Years of education, median (IQR)	14 (12–16)	12 (9–14)	0.001
Married, *n* (%)	54 (9.7)	14 (24.6)	0.001
Employed, *n* (%)	281 (50.7)	17 (29.3)	0.002
Duration of symptoms (months) before diagnosis, median (IQR)	2 (1–6)	6 (3–12)	0.001
Days from -first medical contact to HIV diagnosis, median (IQR)	10 (0–62.5)	52 (0–182)	0.007
Number of consults before diagnosis, median (IQR)	1 (1–2)	2 (1–3)	0.001
Advanced HIV disease at enrollment, *n* (%)	237 (42.2)	32 (55.2)	0.01

IQR, interquartile range.

Women experienced a longer median duration of symptoms before HIV diagnosis [6 months [IQR 3−12] vs. 2 months [IQR 1−6], *p* = 0.001] and a significantly longer time from first medical contact to diagnosis [median 52 days [IQR 0−182] vs. 10 days [IQR 0−62.5], *p* = 0.007] (Table 1).

**Figure 2:**
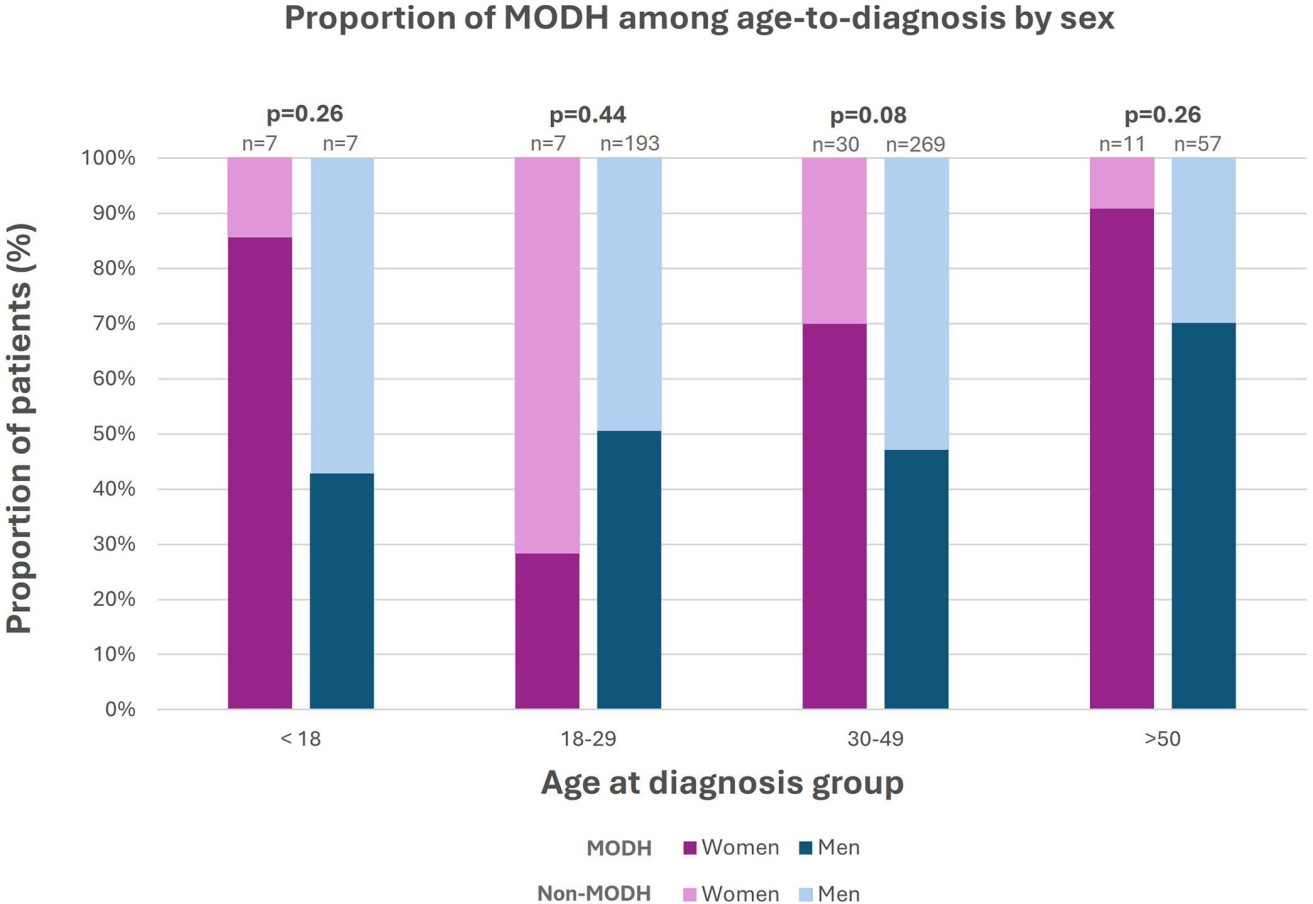
Proportion of patients with and without MODH across age-at-diagnosis by sex. MOHD, missed opportunities for HIV diagnosis.

Among the 320 patients with MOHD, 115 (36%) presented advanced HIV disease criteria at diagnosis, and it was more frequent among women compared to men (51.3% vs. 33.8% *p* = 0.02) (Table 2). The proportion of patients with and without MOHD was stratified by age group at the moment of diagnosis (Figure 2). Among men, the proportion of MOHD increased with age, ranging from 42.9% in those who received HIV diagnosis under 18 y/o to 70.2% in those over 50. On the other hand, MOHD were most frequent in women diagnosed at age 18 (85.7%) and in those diagnosed after age 50 (90.9%). Across the other age groups, women had higher proportions of MOHD, except in the 18–29 group, where men had more MOHD (50.8% vs. 28.6%). However, none of these differences were statistically significant (Fisher's exact test *p* = 0.26, 0.44, 0.08, 0.26).

**Table 2:** Basal characteristics MOHD vs. Non-MOHD by sex.

Variable	All (*n* = 619)		Non-MOHD (*n* = 261)		MOHD (*n* = 320)	
	Men (*n* = 561)	Women (*n* = 58)	Men (*n* = 245)	Women (*n* = 16)	Men (*n* = 281)	Women (*n* = 39)
Age in years at diagnosis, median (IQR)	32 (42–27)	37 (48–27)	31 (38–26)	33.5 (45.7–27)	34 (44–27)	39 (50–31)
Years of education, median (IQR)	14 (16–12)	12 (14–9)	14 (16–12)	11.2 (12.5–7.5)	14 (16–11.7)	12 (15.2–9)
Married, *n* (%)	54 (9.6)	14 (24.6)	21 (8.6)	5 (31.3)	30 (10.7)	8 (20.5)
Advanced HIV disease, *n* (%)	212 (37.8)	27 (46.6)	96 (39.2)	5 (31.3)	95 (33.8)	20 (51.3.)

MOHD, missed opportunities for HIV diagnosis; IQR, interquartile range.

The results of the multivariate regression model are shown in Table 3. In the analysis, increasing age was associated with a higher likelihood of MOHD (OR 1.01, 95% CI 1.00−1.1, *p* = 0.02). Being a woman at birth was also significantly associated with MOHD (OR 2.3, 95% CI 1.21−4.52, *p* = 0.01). Employment status (OR 1.18, 95% CI 0.84−1.68, *p* = 0.32), married (OR 0.91, 95% CI 0.58−1.82, *p* = 0.91), years of education (OR 0.99, 95% CI 0.98−1.004, *p* = 0.21) and duration of symptoms (OR 1.06, 95% CI 0.99−1.01, *p* = 0.26) were not significantly associated with a diagnostic delay in the multivariate analysis.

**Table 3:** Multivariate logistic regression assessing factors related to MOHD.

Variable	OR (CI 95%)	*p* value
Age (years)	1.01 (1.00–1.1)	0.02
Sex at birth (woman)	2.3 (1.21–4.52)	0.01
Education (years)	0.99 (0.98–1.00)	0.21
Marriage status (yes)	0.91 (0.58–1.82)	0.91
Employed (yes)	1.18 (0.84–1.68)	0.32
Time of symptoms (months)	1.06 (0.99–1.01)	0.26

OR odds ratio; CI, confidence interval.

## Discussion

Our study revealed the proportion of MOHD in our newly admitted HIV patients, with a significant sex disparity, being women who experienced higher rates of delayed diagnosis compared to men. More than two-thirds of the women and half of the men population who sought medical care for HIV-related symptoms had at least one MOHD. Additionally, women experienced longer diagnostic delays than men, both from symptom onset and from the first medical contact to HIV diagnosis.

Few studies have comprehensively assessed MOHD beyond key populations or individuals with advanced disease. A cross-sectional study in France highlighted persistently high rates of MOHD in their cohort, although it predominantly focused on MSM, limiting its applicability to a broader demographic, ultimately overlooking the specific challenges faced by women (25). In contrast, our findings contribute to this evidence by showing that nearly half of individuals with MOHD were diagnosed with advanced HIV, and that women in particular experienced greater diagnostic delays than men.

Furthermore, a large U.S. cohort study that stands out as one of the few to analyze MOHD specifically in women, reported that 73% had advanced HIV at diagnosis and 79% developed AIDS within a month (26), highlighting the severe consequences of delayed recognition. Compared with our findings, the higher proportion of MOHD reported by Duffus et al. may be partly explained by differences in study design. Their analysis covered a 12-year period (1996–2007), which is comparable to ours (2013–2023; 10 years), but included a larger cohort size and a broader definition of missed opportunities. In their study, any medical encounter was considered a potential testing opportunity, regardless of its relevance to HIV-related symptoms. In contrast, our study applied a more specific definition, counting only visits prompted by symptoms suggestive of HIV infection, and additionally provides a direct comparison between men and women within a Mexican tertiary care setting.

While socio-demographic characteristics have been previously described to assess differences in HIV risk in Mexico (27), our work specifically focuses on highlighting the sex-based disparities in diagnostic delays, healthcare-seeking patterns, and clinical outcomes. Within this framework, a multicenter study in Mexico highlighted how national screening programs primarily target high-risk groups, leaving women largely excluded from routine HIV testing (12). In that same study, Martin-Onraët et al. described that nearly half of the women were diagnosed with severe immunosuppression and that those who sought medical care three or more times for HIV-related symptoms without being tested had CD4 counts below 200 cells/mm^3^, resulting in a higher incidence of opportunistic infections and worse outcomes. The authors identified several vulnerability factors such as low educational attainment, low income, experiences of violence, early pregnancy, low condom use, and acquiring HIV through stable partners that help contextualize the disparities observed in our cohort.

Beyond reaffirming these barriers, our study reveals that diagnostic delays remain disproportionately greater among women by comparing MOHD between sexes. Additionally, our findings provide updated evidence that underscores the persistence of structural inequities in HIV testing and diagnosis in Mexico. Current national guidelines recommend universal HIV testing during pregnancy, while screening strategies for women outside this context or for individuals not considered part of key populations remain limited (8, 28). Likewise, the most recent version of CENSIDA's *Guidelines for Antiretroviral Treatment in people living with HIV* emphasizes prevention strategies as pre- and post-exposure prophylaxis among key populations, without providing explicit recommendations for their use beyond those groups (29). This approach could help explain our finding that nearly two-thirds of women in our cohort who actively sought care for HIV-related symptoms still experienced MOHD in a higher proportion than men.

Factors such as experiences of violence, early pregnancy, low educational attainment, low income, low condom use, and acquiring HIV through stable partners were not assessed in our study, but were associated with late diagnoses in a previous study from Mexico (12). Additionally, older age may be associated with a lower perception of HIV risk, particularly among women over 40, and social factors such as unemployment (30) and HIV related stigma, which in Latin America is often stronger for women (31), can also delay testing and care. These vulnerabilities may partly explain the disparities observed in our cohort, however, a direct correlation cannot be established due to the retrospective nature of our study; therefore, we recommend to assess these variables in order to have more information.

In addition, although our dataset does not specify whether prior medical visits were with general practitioners or other specialists, previous studies have described an association between primary care encounters and missed opportunities for HIV diagnosis (20). In those settings, clinicians may be less likely to offer HIV testing unless patients explicitly request it, or they may fail to consider HIV infection in women who do not fit a high-risk profile. While we cannot determine if the provider of prior medical visits was a specialist or a general practitioner, the patterns of delayed diagnosis observed in our cohort are consistent with these previously reported barriers. Greater efforts are therefore needed to integrate HIV screening into routine health care visits, particularly in primary care settings (12, 15, 32). This could include the implementation of routine opt-out testing, which has been shown to reduce missed opportunities compared with clinician-initiated testing in emergency settings (32).

Social determinants of health, such as unemployment, marital status, and education, also showed varying patterns among women in our study. These factors have been consistently associated with health disparities, as lower education can limit health literacy and awareness of HIV risks (31), while socio-economic barriers such as unemployment or lack of partner support can hinder access to testing and treatment (33). Furthermore, these determinants should be carefully considered when designing HIV prevention strategies and public health campaigns, particularly for marginalized populations.

Cultural factors unique to Latin America, such as machismo, play a role in perpetuating sex inequality and increasing women's vulnerability to HIV (34). Machismo can not only undermine women's autonomy in sexual decision-making but also promote risk-taking behaviors in men, which can contribute to a higher incidence of HIV transmission to their partners. Programs that encourage open discussions about sexual health, empower women to take control of their sexual and reproductive health, and challenge harmful sex norms could help reduce missed diagnoses and improve prevention efforts in the region (33, 34).

Several strategies have been described to help reduce missed opportunities for HIV diagnosis among women. For example, routine opt-out HIV testing, in which HIV testing is performed unless the patient declines, has been shown to increase testing uptake and reduce missed diagnoses and is endorsed by the Centers for Disease Control and Prevention (35). In addition, expanding HIV testing for women beyond pregnancy-related care, including routine testing during primary care, internal medicine, and gynecology visits across the life course, may help avoid missed diagnoses once pregnancy-based screening ends (36). Finally, studies focused on women have shown that offering HIV testing during healthcare encounters commonly attended by women, regardless of perceived risk or reproductive status, may reduce diagnostic delays and missed opportunities (26).

This study has several limitations, primarily its retrospective nature, which may introduce recall bias or incomplete documentation regarding prior medical visits. Our population only included patients within our tertiary-care center, thus further considerations may be needed to extrapolate this study to the primary care setting. Additionally, the questionnaire evaluated with open-ended questions the symptoms for which the patients sought medical attention. Although we did not assess specific predefined symptoms, it is applied by infectious disease specialists who are trained to suspect and diagnose symptoms that should prompt HIV testing.

Furthermore, due to the retrospective design, we could not determine the clinical appropriateness of HIV testing for each healthcare encounter prior to diagnosis. Nevertheless, using self-reported healthcare visits preceding HIV diagnosis represents a pragmatic approach commonly used in retrospective studies to identify potential missed opportunities (12).

Moreover, our analysis focuses on missed opportunities from a healthcare provider perspective, emphasizing diagnostic delays within the medical system. Within this framework, even if time from symptom onset to HIV diagnosis was evaluated, this information was obtained retrospectively through patient report and is subject to inaccuracies in patient recall. Therefore, patient delay could only be assessed in a limited manner, and the analysis primarily reflects provider- and system-level missed opportunities occurring after healthcare contact. In parallel, other factors that remain unexplored in women living with HIV are limited support networks, food insecurity, and intimate partner violence, which can create barriers to accessing prevention and care and add to the stigma surrounding HIV diagnosis.

Although differences in the prevalence of advanced HIV disease were observed across sex and age groups among patients with MOHD, these results should be interpreted with caution. These analyses were descriptive in nature and did not evaluate associations between MOHD and clinical outcomes such as advanced HIV disease. Consequently, the observed differences reflect prevalence patterns, rather than evidence of causality, and potential confounding factors were not accounted for. Also, the number of women included in the study was small (*n* = 39). As a result, the observed association between female sex and MOHD should be interpreted with caution. Larger studies with greater representation of women are needed to confirm these findings and characterize sex specific factors associated with delayed HIV diagnosis.

Overall, our findings, beyond showing that women are more frequently diagnosed at advanced stages, suggest that there may be structural and provider-related barriers even when women seek medical care. Overcoming these gaps will require coordinated efforts between health authorities and health providers to integrate HIV testing into primary care and to ensure adherence to screening guidelines and recommendations. This means offering HIV testing to all individuals once they become sexually active, regardless of sex, number of sexual partners or belonging to a key population. Additionally, public policies must address the social and cultural factors that delay testing among women, to reduce disparities in HIV diagnosis and to promote more equitable health outcomes.

## Conclusion

In our cohort, women with HIV face significantly higher rates of missed opportunities for diagnosis compared to men, often despite actively seeking medical care for HIV-related symptoms. They experienced longer delays, attended more medical consultations, and presented more frequently with advanced disease. These patterns may reflect broader issues: low perception of risk for HIV, less access to testing, prevention strategies and social barriers, which warrant further research. HIV screening policies must move beyond key population models and include intersectional approaches that recognize the unique sociocultural and clinical barriers women face in our region.

## Data Availability

The raw data supporting the conclusions of this article will be made available by the authors, without undue reservation.
